# Phytotherapy and the Role of Bioactive Compounds in Modulating Mechanisms of Overweight and Obesity Comorbid with Depressive Symptoms—A Scoping Review of Mechanisms of Action

**DOI:** 10.3390/molecules30132827

**Published:** 2025-06-30

**Authors:** Klaudia Sochacka, Sabina Lachowicz-Wiśniewska

**Affiliations:** Faculty of Medicine and Health Science, University of Kalisz, pl. W. Bogusławskiego 2, 62-800 Kalisz, Poland; szd.5.2023@uniwersytetkaliski.edu.pl

**Keywords:** phytotherapy, bioactive compounds, obesity, depression, polyphenols, neuroinflammation, gut–brain axis, antioxidant mechanisms

## Abstract

Obesity and depression frequently coexist, sharing overlapping molecular pathways such as inflammation, oxidative stress, gut microbiota dysbiosis, and neuroendocrine dysfunction. Recent research highlights the therapeutic potential of plant-derived bioactive compounds in targeting these shared mechanisms. This scoping review followed Preferred Reporting Items for Systematic Reviews and Meta-Analyses (PRISMA) guidelines and included 261 peer-reviewed studies identified through PubMed, Scopus, and the Web of Science up to December 2024. Studies were screened based on predefined inclusion and exclusion criteria. This review synthesizes data from peer-reviewed studies, including both preclinical and clinical investigations, focusing on polyphenols, flavonoids, alkaloids, and other phytochemicals with anti-inflammatory, antioxidant, neuroprotective, and metabolic effects. Compounds such as quercetin, epigallocatechin gallate (EGCG), resveratrol, curcumin, anthocyanins, and luteolin demonstrate promise in modulating adenosine monophosphate-activated protein kinase (AMPK), brain-derived neurotrophic factor (BDNF), nuclear factor kappa B (NF-κB), and gut–brain axis pathways. Our scoping review, conducted in accordance with PRISMA guidelines, identifies promising combinations and mechanisms for integrative phytotherapy. These findings underscore the potential of botanical strategies in developing future interventions for metabolic and mood comorbidities.

## 1. Introduction

Obesity remains one of the most critical and escalating global public health concerns. Despite extensive research efforts, effective treatment strategies are still limited, and the global prevalence of obesity continues to rise at an alarming rate [1]. Obesity is defined as the excessive accumulation of adipose tissue resulting from a sustained positive energy balance, which contributes to the development of various metabolic and/or inflammatory disorders [2]. This condition poses a substantial health burden on modern populations. A significant complication is its frequent coexistence with depression, a condition often referred to as a “silent pandemic” due to its complex social and health consequences. Both conditions are associated with high morbidity and mortality, affecting millions of individuals globally.

Depression is the most common mental illness, characterized by symptoms such as persistent low mood, anhedonia, feelings of guilt, impaired concentration, low self-esteem, sleep disturbances, and appetite dysregulation—either hyperphagia or hypophagia [3]. According to a meta-analysis by Luppino et al. [4], a bidirectional association exists between obesity and depression: each condition increases the risk of developing the other. Obesity may predispose individuals to depression, whereas depression can act as a risk factor for the onset of obesity [4].

In the search for new therapeutic strategies to address both obesity and depression, increasing attention has been directed toward plant-derived materials. Owing to their high content of bioactive phytochemicals, plant-derived substances have emerged as promising adjunctive agents in the treatment of both conditions. Research indicates that certain plant-based compounds may mitigate chronic inflammation, thereby supporting patient health. Among the most promising bioactive compounds with anti-obesity potential are polyphenols (such as resveratrol, quercetin, and epigallocatechin gallate), betalains, omega-3 fatty acids, and dietary fiber [5].

Depression is a condition in which inflammatory processes can significantly exacerbate clinical symptoms. Inflammation may represent not only a physiological manifestation of depression but also a potential etiological factor [6]. Therefore, the anti-inflammatory and antioxidant properties of natural bioactive compounds, as with obesity, are of considerable importance in the supportive management of depression. Several plant-derived compounds have demonstrated antidepressant-like effects in preclinical and clinical models, including apigenin, berberine, curcumin, genistein, luteolin, N-acetylcysteine, naringenin, piperine, quercetin, resveratrol, epigallocatechin gallate, and baicalin [7].

This review aims to critically evaluate the current scientific evidence on the health-promoting potential of selected plant-derived bioactive compounds in the prevention and adjunctive treatment of obesity and depression. Emphasis is placed on molecular and cellular mechanisms, including anti-inflammatory, antioxidant, neuroprotective, and metabolic pathways. Both in vitro and in vivo studies are included to provide a comprehensive overview of the dual-action phytotherapeutic approach.

## 2. Materials and Methods

**Figure 1:**
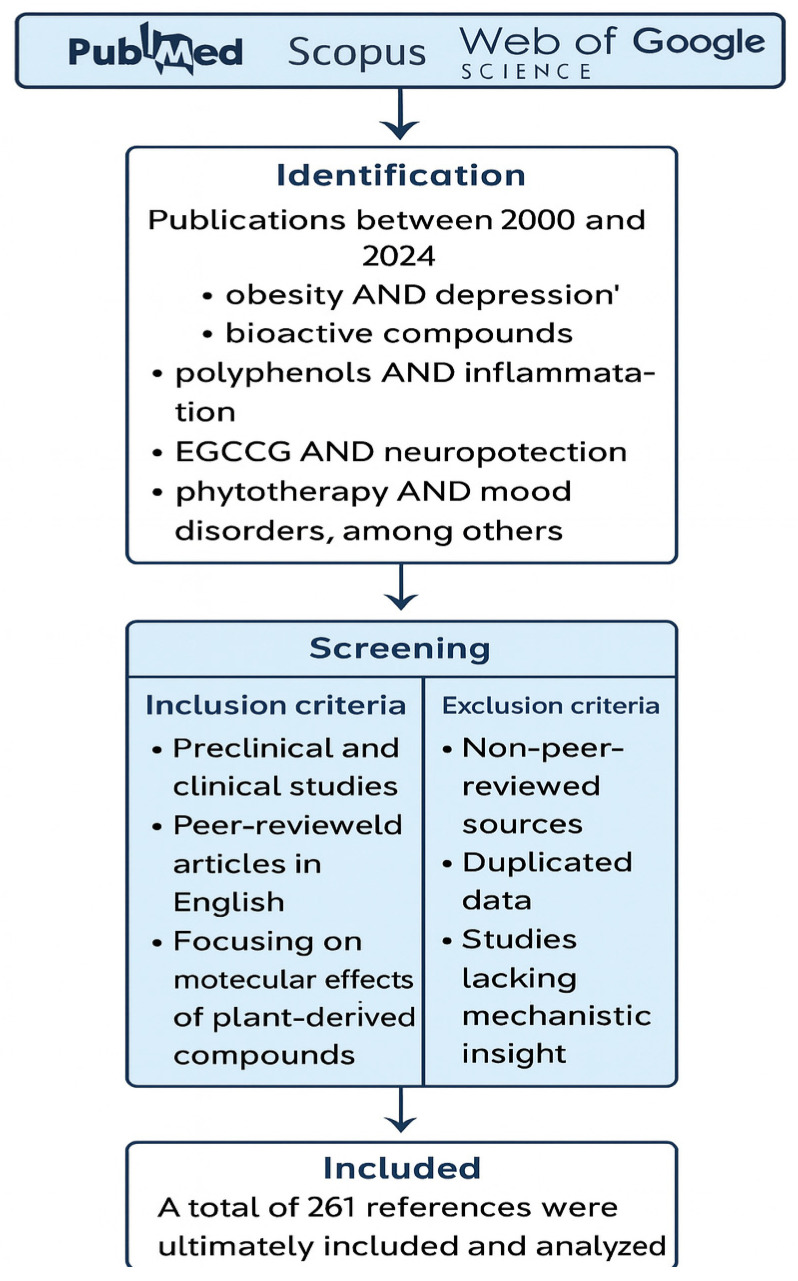
PRISMA-ScR flow diagram illustrating the study selection process. After the removal of duplicates and the application of inclusion and exclusion criteria, 261 publications were included in the final analysis.

This review was conducted following PRISMA guidelines for scoping reviews. A structured literature search was performed using PubMed, Scopus, Web of Science, and Google Scholar databases for publications published between January 2000 and December 2024 (Figure 1). The search strategy included the following keywords and their combinations: “obesity and depression,” “bioactive compounds,” “polyphenols and inflammation,” “curcumin and neuroprotection,” “EGCG and metabolism,” and “phytotherapy and mood disorders.” Boolean operators were used to refine the search, and Medical Subject Headings (MeSH) terms were applied where appropriate.

The final search was conducted on 20 December 2024. The search results were imported into EndNote for duplicate removal, and studies were screened based on titles and abstracts, followed by full-text evaluation. Two independent reviewers conducted the selection process. Discrepancies were resolved through discussion or, if needed, consultation with a third reviewer.

Inclusion and exclusion criteria were applied as follows: (i) included: peer-reviewed articles in English focusing on plant-derived bioactive compounds with mechanistic or interventional relevance to obesity and/or depression, including both preclinical (in vitro and in vivo) and clinical studies; (ii) excluded: non-peer-reviewed articles, review articles, case reports, editorials, conference abstracts, studies without mechanistic data, and duplicates.

A total of 261 references were ultimately included and analyzed. Risk of bias was not formally assessed due to the exploratory nature of the scoping review. This review was not registered in PROSPERO or any other platform.

Data extraction was performed using a standardized form that included study type (in vitro, in vivo, clinical), bioactive compound (e.g., curcumin, EGCG, resveratrol), mechanism of action (e.g., modulation of AMPK, BDNF, NF-κB), health outcome (e.g., anti-inflammatory, antioxidant, antidepressant, anti-obesity), and other relevant parameters. Extracted data were synthesized qualitatively.

The final pool of articles (*n* = 261) was analyzed narratively with particular attention to biochemical pathways, pharmacological targets, and the potential synergistic effects of bioactive compounds in the context of obesity and depression comorbidity. Among the included studies, approximately 45 were in vitro studies, 70 were conducted in vivo using animal models, and 55 were clinical investigations (observational and interventional). The most frequently studied phytochemicals were polyphenols, including quercetin, resveratrol, epigallocatechin gallate (EGCG), curcumin, anthocyanins, and luteolin. These compounds were shown to exert antioxidant, anti-inflammatory, neuroprotective, and metabolic effects, often through the modulation of AMPK, NF-κB, BDNF, the hypothalamic–pituitary–adrenal (HPA) axis, and gut microbiota. Approximately 100 studies addressed anti-inflammatory effects, 60 explored antioxidant mechanisms, 40 focused on neuroprotective properties, and 61 examined anti-obesity effects. The reviewed data highlight the multidimensional potential of plant-derived bioactive compounds in targeting interrelated pathophysiological pathways in metabolic and mood disorders.

## 3. Epidemiological and Public Health Impact of Obesity and Depression

According to the Global Burden of Disease study [1], obesity and depression rank among the most significant global health challenges. Their co-occurrence poses a serious threat to patient health and well-being [8]. Both obesity and depression are often described as epidemics of the 21st century and constitute significant public health concerns, particularly due to the escalating costs associated with their treatment. In recent years, greater attention has been directed toward the psychological implications of obesity, including low self-esteem, body image dissatisfaction, and social withdrawal. These psychosocial consequences frequently contribute to chronic stress, emotional dysregulation, and, ultimately, the onset of depressive symptoms. These psychosocial issues may lead to chronic stress, a persistently low mood, and ultimately, the development of depression.

Conversely, depression—which is characterized by persistent sadness, anhedonia, and stress vulnerability—may promote maladaptive eating behaviors such as emotional overeating or food restriction. These behaviors often serve as coping mechanisms for psychological distress and can lead to or exacerbate obesity [9]. Globally, the prevalence of both conditions continues to escalate. According to the World Health Organization (WHO), the global prevalence of obesity has nearly tripled since 1975, while over 300 million individuals are currently affected by mental health disorders, with depression being one of the most commonly diagnosed psychiatric conditions [10].

In the Polish context, national surveillance data reveal a disturbing upward trend in obesity rates, particularly among children and adolescents, positioning Poland among the leading European countries in this regard [11]. The growing incidence of both obesity and depression contributes significantly to the burden of non-communicable diseases (NCDs), especially in high-income and rapidly developing nations. The treatment and management of patients suffering from comorbid obesity and depression generate considerable strain on healthcare resources. These burdens include extended treatment durations, frequent relapses, reduced treatment efficacy, and increased incidence of comorbid complications, such as cardiovascular disease, type 2 diabetes, and anxiety disorders, further exacerbating public health and economic challenges [12,13].

## 4. The Role of Major Classes of Bioactive Compounds in the Treatment of Obesity and Depression

Plant-derived bioactive compounds—including polyphenols, phenolic acids, polysaccharides, saponins, omega-3 fatty acids, and probiotics—have shown therapeutic potential in obesity and depression due to their ability to modulate inflammation, oxidative stress, lipid metabolism, and the gut–brain axis. Their mechanisms involve the inhibition of adipogenesis, the regulation of transcription factors (e.g., PPARγ and C/EBPα), the suppression of pro-inflammatory cytokines (e.g., TNF-α and IL-6) via NF-κB inhibition, and neurochemical modulation [14,15,16,17,18,19].

Polyphenols are the most abundant phytochemicals in the human diet and are recognized for their anti-obesity and neuroprotective effects. Compounds such as quercetin, resveratrol, curcumin, and catechins inhibit adipogenesis and lipogenesis, stimulate fatty acid oxidation, and promote adipocyte apoptosis [14,15,16,17,18,19]. Hsu and Yen [20] demonstrated that rutin significantly reduces intracellular triglycerides and downregulates key adipogenic factors while increasing adiponectin levels, thereby supporting improved lipid profiles. Additionally, polyphenols enhance antioxidant enzyme activity and reduce oxidative stress associated with adiposity and neuroinflammation [20].

Phenolic acids also exert strong antioxidant and anti-inflammatory properties. In vitro studies show that they suppress macrophage infiltration and reduce cytokines such as MCP-1 and PAI-1 via NF-κB inhibition, while modulating adipokine secretion [21,22,23]. Their ability to maintain a redox balance makes them valuable agents in preventing chronic metabolic and mood disorders.

Polysaccharides are complex carbohydrates with immunomodulatory and anti-inflammatory properties. Their consumption influences the regulation of the gut microbiota, which is crucial for both metabolic and mental health. Polysaccharides can increase satiety, which promotes weight control, while at the same time they induce beneficial changes in the nervous system, alleviating symptoms of depression by affecting the brain-gut axis and modulating neurotransmitters [24,25,26,27,28,29,30,31,32,33]. Zou et al. [34] reported that insoluble fiber enhances IL-22 expression, contributing to intestinal barrier integrity and microbial defense. In contrast, a study by Sanchez et al. [35] indicates that soluble fiber intake may increase obesity-associated inflammation because the fiber helps protect tissues from the oxidative stress characteristic of the disease [36]. Moreover, studies by Ma et al. [36] confirmed that both soluble and insoluble fibers reduce CRP and LPS levels, thus lowering systemic inflammation and improving metabolic parameters [34,35,36,37,38].

Omega-3 fatty acids (such as EPA and DHA) contribute to the resolution of inflammation through the activation of PPARγ and the inhibition of NF-κB, which reduces pro-inflammatory cytokine levels and improves insulin sensitivity. Probiotic strains, particularly *Lactobacillus* and *Bifidobacterium*, enhance gut microbial balance, reduce intestinal permeability, and decrease LPS-induced inflammation. Their synergistic effect with bioactive plant compounds further supports their use in the management of metabolic and neuropsychiatric conditions [39,40].

In summary, different classes of natural bioactive compounds modulate shared pathophysiological pathways involved in obesity and depression. Through combined anti-inflammatory, antioxidant, and microbiota-regulating actions, they hold promise as adjunct therapeutic agents. While oral supplementation remains the most common route of administration, further clinical trials are needed to confirm long-term efficacy, optimize dosage, and evaluate safety.

## 5. The Role of Bioactive Compounds in the Management of Obesity and Depression

Although this review primarily focuses on the bioactivity and mechanisms of plant-derived compounds, it is important to note that phytotherapy may be administered via various routes, each potentially influencing efficacy, particularly in mood disorders. While oral delivery (e.g., capsules, teas, tinctures) remains the most common and practical approach, it is often subject to first-pass hepatic metabolism and variable absorption.

Alternative routes—such as inhalation (e.g., aromatherapy with lavender or bergamot oils), transdermal application, or sublingual tinctures—may offer faster onset and bypass hepatic degradation. Intraperitoneal and intravenous administration have also been explored in experimental models to isolate specific mechanistic effects [41,42,43,44].

Most in vivo and clinical studies reviewed here employed oral administration due to its ease and regulatory acceptance. However, future research should evaluate the pharmacokinetics and comparative efficacy of novel formulations, such as nanoemulsions (e.g., nanoencapsulated curcumin), liposomes, and transdermal systems, to improve therapeutic outcomes and patient adherence [45,46,47,48,49].

### The Role and Classification of Bioactive Compounds in Therapy

Nature offers an almost limitless reservoir of compounds with potential health benefits, commonly referred to as natural bioactive substances. Bioactive compounds are naturally occurring substances that can exert favorable effects on human health. In the context of nutrition, they have been described as nutraceuticals since 1979, as their consumption provides health benefits that extend beyond basic nutritional needs [50]. These valuable substances can be classified into four main categories: macronutrients, micronutrients, phytochemicals, and regulators of the gut microbiota [51].

Macronutrients include carbohydrates, fats, and proteins. Micronutrients comprise essential vitamins and minerals that are indispensable for proper physiological functioning. Phytochemicals include diverse groups such as terpenes, alkaloids, and phenolic compounds. Lastly, the microbiota-regulating group includes probiotics, prebiotics, synbiotics, and postbiotics [52].

**Figure 2:**
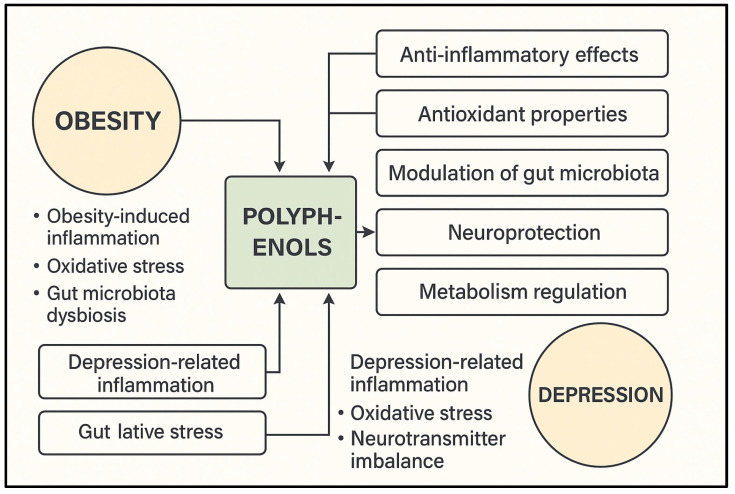
Bidirectional pathophysiological links between obesity and depression mediated by inflammation, oxidative stress, and gut dysbiosis. Polyphenolic compounds counteract these shared mechanisms through anti-inflammatory, antioxidant, microbiota-modulating, neuroprotective, and metabolic effects.

This review specifically focuses on phenolic compounds—especially polyphenols—due to their demonstrated efficacy in targeting inflammation, oxidative stress, metabolic dysregulation, and neurochemical imbalances that are commonly shared between obesity and depression (Figure 2).

## 6. Selected Bioactive Compounds and Their Mechanisms of Action

Obesity and depression share overlapping inflammatory, metabolic, and neuroendocrine pathways. Chronic low-grade inflammation, which is characterized by the increased secretion of pro-inflammatory cytokines (TNF-α, IL-6, and MCP-1), dysregulated adipokine profiles (leptin, resistin, and adiponectin), and oxidative stress, contributes to both conditions. Several plant-derived compounds, particularly polyphenols, modulate key molecular targets, such as AMPK, NF-κB, PPARγ, and BDNF, thereby improving metabolic homeostasis and CNS function. Below, selected phytochemicals are discussed with emphasis on their dual activity.

### 6.1. The Treatment of Obesity and Its Mechanisms of Action

Excessive adipose tissue expansion in obesity promotes chronic low-grade inflammation, largely through the dysregulated secretion of adipokines and pro-inflammatory cytokines, such as TNF-α, IL-6, MCP-1, resistin, and visfatin [53,54,55,56]. This inflammatory milieu, exacerbated by the increased production of ROS and RNS, contributes to metabolic disturbances and comorbidities like type 2 diabetes and cardiovascular disease [57] (Table 1). Bioactive compounds may counteract these effects by modulating key molecular pathways, including PPARα, COX-2, GLUT4, SIRT1, and PGC1-α, thereby improving energy metabolism and inflammatory balance [5,58].

Polyphenols are prominent dietary phytochemicals that are found in fruits, vegetables, tea, and whole grains [59,60,61,62]. They include flavonoids, phenolic acids, stilbenes, and curcuminoids, and exhibit anti-obesity activity by suppressing adipogenesis and lipogenesis, inducing adipocyte apoptosis, and downregulating lipogenic genes such as PPARγ and SREBP1 [63,64,65]. Polyphenols also support gut microbiota modulation and reduce inflammatory cytokine expression, contributing to systemic metabolic regulation [66]. Compounds such as quercetin, resveratrol, EGCG, and genistein primarily inhibit adipocyte differentiation, positioning them as promising agents for obesity prevention and body weight control [67].

Quercetin, a flavonoid that is abundant in onions, garlic, apples, and citrus fruits, is recognized as one of the most potent plant-derived antioxidants. Although numerous in vitro and in vivo studies confirm its anti-obesity potential, human clinical data remain limited [67,68,69]. In vitro, quercetin inhibits adipogenesis in preadipocytes and reduces inflammatory cytokine secretion in macrophages and adipocytes exposed to TNF-α [70,71,72,73]. It also improves insulin sensitivity in inflamed adipocytes [72,74,75].

A clinical trial using 150 mg/day quercetin supplementation in overweight individuals with different ApoE genotypes demonstrated reduced waist circumference and triglyceride levels [76]. In vivo, quercetin inhibited adipogenic marker expression in F344 rat muscle progenitor cells [77]. Beyond metabolic benefits, it also exhibits antidepressant-like effects by lowering plasma cortisol, improving memory, and reducing anxiety- and depressive-like behavior [78,79,80], potentially through the modulation of BDNF and iNOS.

Genistein, an isoflavone found in soy, has demonstrated anti-obesity activity through the modulation of lipid metabolism and energy balance [81]. Mice supplemented with 600 mg or 1500 mg/kg genistein showed significant weight loss, improved glucose and lipid profiles, and enhanced adipocyte apoptosis [82,83]. Additionally, 0.2% genistein included in a high-fat diet improved glucose uptake and reduced hepatic steatosis [84]. These effects are linked to increased lipolysis, fatty acid oxidation, and the upregulation of lipid metabolism genes [85].

Emerging studies have suggested that genistein may also exert antidepressant-like effects, possibly via serotonergic 5-HT1A receptor interaction and the inhibition of monoamine oxidase (MAO), which enhances brain serotonin, dopamine, and norepinephrine levels [86,87,88,89]. In vivo, genistein’s efficacy was comparable to amitriptyline at 10 mg/kg [88]. Given these findings, genistein is a promising nutraceutical candidate—e.g., standardized soy isoflavone supplements (30–50 mg/dose)—with the potential for bioavailability-enhanced formulations. Future clinical trials should target overweight adults with insulin resistance and assess both metabolic and inflammatory endpoints.

Resveratrol, a polyphenol that is highly concentrated in red grape skins, is also found in tea, berries, pomegranate, nuts, and dark chocolate [90]. It possesses strong anti-inflammatory and antioxidant properties and is known to activate sirtuin 1 (SIRT1), a regulator of mitochondrial function and cellular metabolism [91]. Resveratrol also modulates AMP-activated protein kinase (AMPK), thereby promoting energy balance and lipid homeostasis.

Through AMPK activation, resveratrol inhibits adipocyte differentiation and lipogenesis while promoting lipolysis and β-oxidation [92]. In vitro, it enhances apoptosis in mature adipocytes and reduces fat accumulation [93,94,95]. In vivo rodent studies confirm reductions in fat mass via the suppression of lipid deposition and the stimulation of oxidative pathways [96,97].

Beyond metabolic effects, resveratrol exhibits neuroprotective activity. It elevates dopamine and serotonin levels in the prefrontal cortex and upregulates neuropeptide Y (NPY), thereby influencing mood, appetite, and stress responses [98,99]. Studies in corticosterone-induced depression models demonstrate that resveratrol improves mood by modulating the HPA axis and increasing hippocampal BDNF levels [100]. Additional evidence suggests that it promotes neurogenesis, regulates SIRT1, and suppresses NF-κB signaling, collectively contributing to its antidepressant effects [101].

Epigallocatechin gallate (EGCG), the primary catechin in green tea, exerts potent anti-obesity effects by influencing both nutrient absorption and intracellular energy regulation. One of its main molecular targets is AMP-activated protein kinase (AMPK), which is a central regulator of cellular energy homeostasis. EGCG activates AMPK in the liver, skeletal muscle, and adipose tissue by increasing the AMP/ATP ratio, thereby promoting fatty acid oxidation and inhibiting lipogenesis [102].

AMPK activation by EGCG results in the phosphorylation of acetyl-CoA carboxylase (ACC), the inhibition of lipogenic enzymes (acc1, fas, scd1), and the downregulation of transcription factors, such as C/EBPβ, PPARγ, and SREBP1 [102,103,104]. Simultaneously, it enhances lipolysis by upregulating hormone-sensitive lipase (HSL) and related enzymes, and promotes mitochondrial biogenesis via PGC-1α expression, contributing to increased energy expenditure [103].

EGCG also exhibits anti-inflammatory properties by suppressing IL-1β and TNF-α production through the inhibition of the NF-κB and MAPK pathways [105]. Its neuroprotective effects include the modulation of the HPA axis, the regulation of neurosteroid biosynthesis, and the protection of hippocampal DNA from oxidative and apoptotic damage [106].

In a study by Li et al. [107], HFD-fed mice treated with EGCG (50–100 mg/kg/day, 20 weeks) showed significant reductions in body and epididymal fat mass, improved lipid profiles (including lower TG, cholesterol, HDL-C, and LDL-C), and increased fecal excretion of fatty acids. Collectively, EGCG promotes weight loss and metabolic balance through AMPK activation, lipogenesis inhibition, thermogenesis stimulation, and anti-inflammatory and neuroprotective actions [103,105,106,107,108,109].

Anthocyanins, a subclass of phenolic compounds found in purple sweet potatoes, black soybeans, berries, and red fruits, play a central role in regulating energy metabolism and reducing obesity risk. Their key mechanism involves the activation of AMP-activated protein kinase (AMPK), which promotes catabolic processes such as fatty acid oxidation and inhibits lipogenesis and cholesterol synthesis [104,105,106]. Hwang et al. [108] showed that anthocyanins from purple sweet potato activated AMPK in HepG2 cells, thereby reducing hepatic lipid accumulation and body weight in obese mice (200 mg/kg/day). Similar effects were observed with black soybean extract containing cyanidin-3-glucoside [109].

AMPK activation also enhances mitochondrial biogenesis via PGC-1α and upregulates PPARγ, thereby increasing mitochondrial function and reducing adipogenesis [110,111]. Anthocyanins inhibit pancreatic lipase activity, thereby decreasing fat absorption and serum free fatty acids, as shown with lychee flower extract and pomegranate or red orange juices [112]. They also modulate glucose metabolism by inhibiting α-glucosidase and enhancing insulin sensitivity in muscle and liver tissues through AMPK signaling [113].

Moreover, anthocyanins regulate appetite-related hormones. They reduce leptin levels and affect the secretion of ghrelin, CCK, GIP, and GLP-1, thereby enhancing satiety and reducing caloric intake, particularly in individuals with insulin resistance [114,115]. Their prebiotic effects include the stimulation of beneficial microbiota (including Lactobacillus, Bifidobacterium, and Enterococcus), leading to increased short-chain fatty acid production, which further modulates inflammation, appetite, and insulin response [108,109,116,117].

Betalains, which are found in beetroot (*Beta vulgaris*) and dragon fruit (*Hylocereus* spp.), are bioactive pigments with potent antioxidant and anti-inflammatory properties. Betanin, the primary red-violet pigment in beets, reduces ROS, RNS, and proinflammatory cytokines (e.g., TNF-α, IL-1β, and IL-6), as demonstrated by Ahmadi et al. [118]. In a NASH mouse model, Yahaghi et al. [119] found that 20 mg/kg of intraperitoneal betalain administration increased adiponectin and decreased leptin levels, although other studies reported limited effects on lipid profile or body weight [120].

**Figure 3:**
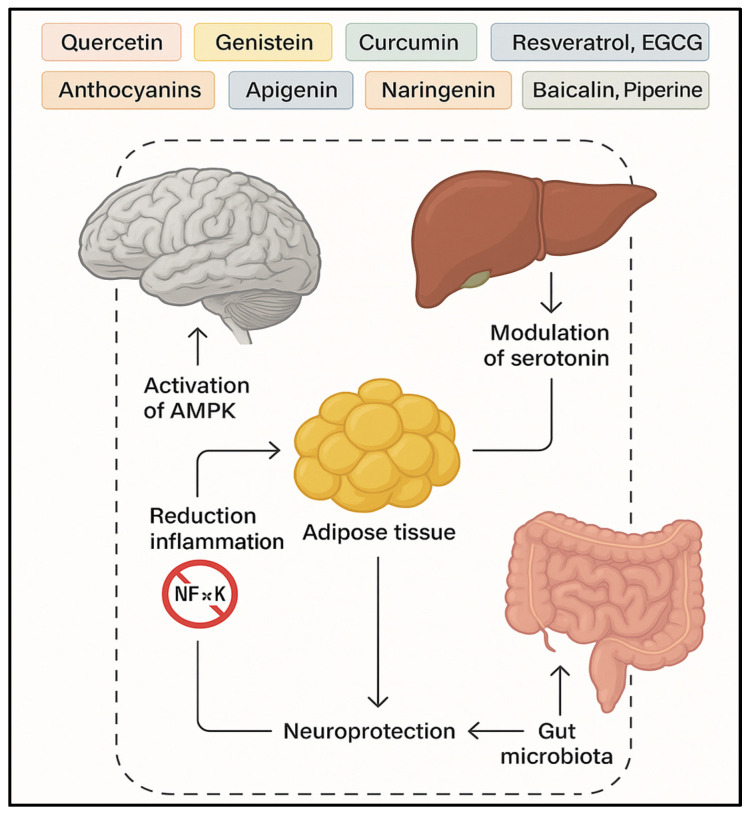
Molecular mechanisms through which selected bioactive compounds (e.g., quercetin, genistein, and curcumin) act on the brain–gut–adipose axis to counteract inflammation, enhance AMPK activity, and modulate serotonin and neuroinflammatory pathways.

In vitro assays confirm betalains’ ROS-scavenging potential using FRAP, ORAC, and ABTS methods. They also inhibit LOX-1, an enzyme involved in ROS generation [121,122,123]. Betalains isolated from quinoa (*Chenopodium quinoa*) further support their antioxidant efficacy, suggesting potential in mitigating oxidative stress-related metabolic disorders (Figure 3) [123,124].

### 6.2. Treatments for Depression Comorbid with Obesity and Their Mechanisms of Action

**Figure 4:**
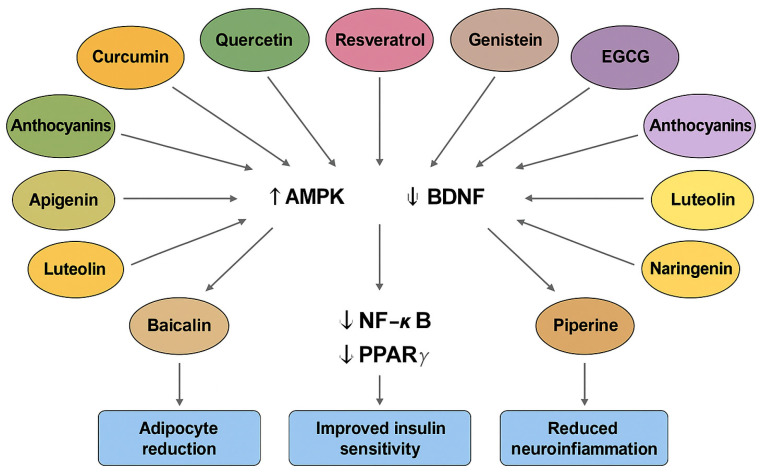
Synergistic actions of plant bioactive compounds on AMPK and BDNF signaling pathways, contributing to adipocyte reduction, improved insulin sensitivity, and neuroprotection.

**Figure 5:**
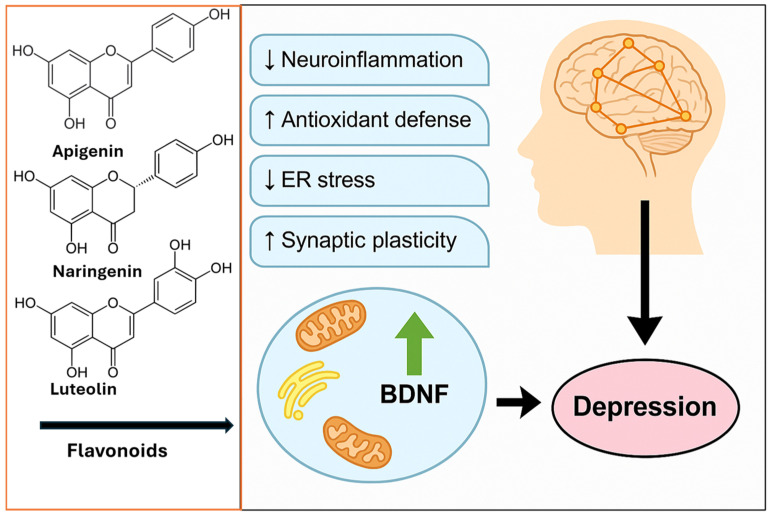
Flavonoid-mediated modulation of neuroinflammation, oxidative stress, and BDNF expression in relation to depressive symptoms.

Building on the general mechanisms introduced earlier, a range of specific plant-derived bioactive compounds has demonstrated therapeutic effects in the context of depression coexisting with obesity. These compounds modulate neurotransmission, attenuate oxidative stress, and influence the hypothalamic–pituitary–adrenal (HPA) axis, thereby contributing to neuroprotection and mood regulation [125,126,127,128,129,130]. Their antidepressant effects are attributed to interactions with monoaminergic systems, the normalization of neurotransmitter levels, and the modulation of inflammatory and neurotrophic signaling pathways (Figure 4 and Figure 5) [131,132].

Apigenin, a flavonoid that is abundant in citrus fruits, exerts antidepressant effects through multiple mechanisms, including the inhibition of inflammatory mediators (iNOS, COX-2), the downregulation of NF-κB signaling, and the activation of neurotrophic pathways, such as p38/MAPK and PI3K/Akt [133,134,135,136,137]. Animal studies have shown increased hippocampal BDNF expression following apigenin administration, highlighting its neuroplasticity-enhancing potential.

Naringenin, another citrus-derived flavonoid, modulates serotonin and norepinephrine levels and restores HPA axis homeostasis. It reduces serum corticosterone, enhances glutathione reductase activity, and decreases pro-inflammatory cytokines (such as IL-1β, IL-6, and TNF-α), while increasing BDNF levels in the hippocampus. Naringenin’s dual modulation of mood and metabolic parameters makes it a promising candidate for mood-support functional beverages (e.g., citrus-based drinks enriched with nanoencapsulated naringenin or citrus peel extracts). Clinical trials could evaluate its synergistic effects when co-administered with selective probiotics or antidepressant therapy in patients with metabolic syndrome and depressive symptoms [138,139,140,141,142,143,144].

Curcumin, the principal curcuminoid from *Curcuma longa*, has been shown to inhibit monoamine oxidase (MAO-A and MAO-B), increase monoamine neurotransmitter levels, and suppress pro-inflammatory signaling (notably via NF-κB). These mechanisms jointly contribute to its antidepressant efficacy demonstrated in both preclinical and clinical models [145,146,147,148,149,150,151,152].

Piperine, the main alkaloid in black pepper, also inhibits MAO enzymes, elevates central monoamine levels, and regulates HPA axis overactivity. Its antidepressant action is further supported by increased BDNF expression and the suppression of oxidative and inflammatory pathways [153,154,155,156,157,158].

Luteolin, which is found in celery, green pepper, and Perilla, crosses the blood–brain barrier and acts as an antioxidant and cytokine inhibitor. It attenuates NF-κB and TLR4 signaling, inhibits monoamine reuptake, and normalizes corticosterone levels, contributing to improved mood and neuroprotection in chronic stress models [159,160,161,162,163,164].

Baicalin, a flavone glycoside with anti-inflammatory and antioxidant properties, activates key intracellular signaling cascades (e.g., AMPK and PI3K/Akt) and enhances neuroplasticity via the TrkB, synaptophysin, and Rac1 pathways [165]. Preclinical studies have shown that baicalin ameliorates depressive-like behaviors in chronic stress models. A meta-analysis of 22 animal studies confirmed its efficacy in standard behavioral paradigms (SPT, TST, FST, and OFT), particularly at doses between 40 and 100 mg/kg [166,167,168,169]. These results point toward baicalin and other flavonoids, such as apigenin or luteolin, as promising candidates for future diet-based interventions or adjunctive nutraceutical therapies. A translational approach may involve testing standardized plant extracts in phase I/II trials with validated psychiatric scales (e.g., HDRS and BDI-II) and inflammatory markers (e.g., IL-6 and CRP) to assess mood stabilization in obese individuals with mild to moderate depressive symptoms [170].

These findings underline the multifaceted roles of bioactive plant compounds in addressing depression co-occurring with obesity through their synergistic anti-inflammatory, antioxidant, neurotrophic, and endocrine-modulating actions (Figure 4, Table 1).

**Table 1:** Key bioactive compounds and their mechanisms.

Compound	Natural Source	Mechanism of Action	References
Quercetin	Onions, apples, and berries	Antioxidant, anti-inflammatory, modulates AMPK and cytokines	[67,68,69,70,71,72,73,74,75,76,77,78,79,80]
Apigenin	Parsley, celery, and chamomile	Anxiolytic, neuroprotective, regulates BDNF, mTOR/AMPK/ULK1	[133,134,135,136,137]
Luteolin	Celery, green pepper, and chamomile	Reduces neuroinflammation, affects ER stress, and monoamine transporters	[159,160,161,162,163,164]
Naringenin	Citrus fruits	Antioxidant, modulates BDNF signaling, antidepressant	[138,139,140,141,142,143,144]
Baicalin	Scutellaria baicalensis	Anti-inflammatory, modulates PI3K/Akt, CREB/BDNF, and BBB protection	[165,166,167,168,169,170]
Genistein	Soy products, soybeans	Phytoestrogenic, affects neurotransmitter systems, regulates lipid metabolism	[81,82,83,84,85,86,87,88,89]
Resveratrol	Grapes, red wine, berries	Activates SIRT1 and AMPK, anti-adipogenic, neuroprotective	[90,91,92,93,94,95,96,97,98,99,100,101]
Curcumin	Turmeric	Anti-inflammatory, modulates NF-κB, serotonin, and dopamine	[145,146,147,148,149,150,151,152]
EGCG	Green tea	Modulates metabolism, anti-obesity, and neuroprotective, insulin sensitivity	[102,103,104,105,106,107]
Anthocyanins	Berries, red cabbage, purple corn	Anti-inflammatory, modulate adipogenesis and lipid metabolism, antioxidant, influence gut-brain axis	[102,103,104,105,106,107]
Betalains	Beetroot, pitaya	Antioxidant, anti-inflammatory, inhibits peroxynitrite	[118,119,120,121,122,123,124]
Piperine	Black pepper	Enhances bioavailability, MAO inhibition, neuroactive	[153,154,155,156,157,158]
Omega-3 fatty acids	Fish oil, flaxseed	Anti-inflammatory, neuroprotective	[170,171,172,173,174,175,176,177,178]

## 7. In Vitro and In Vivo Studies on Bioactive Compounds

Recent studies have highlighted the potential of phytotherapy in modulating mechanisms related to obesity and depression. Below, selected in vitro and in vivo studies are reviewed, focusing on the bioactivity of plant-derived compounds.

### 7.1. In Vitro Studies

In vitro studies have consistently demonstrated that specific bioactive compounds inhibit adipogenesis, lipogenesis, and inflammation in adipocytes. For instance, Chen et al. [171] showed that resveratrol (50 µM) significantly reduced 3T3-L1 preadipocyte differentiation by activating AMPK and downregulating adipogenic markers PPARγ and C/EBPα by 45% and 50%, respectively.

Similar anti-adipogenic effects were observed with curcumin, which attenuated lipid accumulation through NF-κB pathway inhibition and the suppression of pro-inflammatory cytokines [172]. Studies using turmeric extracts confirmed reduced cytokine production in macrophages, supporting its anti-inflammatory potential in obesity-related inflammation [173].

EGCG also inhibited 3T3-L1 adipogenesis by downregulating key transcription factors such as PPARγ and C/EBPα and suppressing lipogenic gene expression [174].

Collectively, these findings support the potential of polyphenolic compounds to reduce adipocyte differentiation and inflammatory responses in vitro, justifying their further evaluation in vivo and in clinical trials.

These data also inform the rational design of multi-compound strategies. The high-throughput screening of polyphenols may facilitate the discovery of synergistic combinations. Ultimately, such insights can guide the development of functional food products—e.g., teas, supplements, or bars—that contain bioavailable formulations of curcumin, EGCG, and quercetin.

### 7.2. In Vivo Studies

In vivo studies play a key role in validating the efficacy of diets enriched with bioactive compounds for managing obesity and depressive symptoms. Several preclinical models have demonstrated dual benefits of natural substances through metabolic and neuroprotective mechanisms.

In a study by Kim et al. [175], turmeric extract (100 mg/kg, 8 weeks) reduced body weight by 15% and serum TNF-α by 35% in HFD-fed rats, with behavioral tests showing improved mood. Similarly, Zhang et al. [176] reported that acai berry extract (200 mg/kg, 6 weeks) decreased body weight (−12%) and cortisol (−30%) while increasing brain serotonin (+25%) in stressed, high-carbohydrate-fed mice.

Khan et al. [177] found that EGCG (50 mg/kg) reduced body weight and visceral fat while improving serotonin and dopamine levels and alleviating depression-like behavior in HFD-fed mice. Omega-3 fatty acids (2 g/kg EPA + DHA) also improved neurochemical and inflammatory profiles—elevating serotonin and reducing IL-6 and TNF-α—alongside benefits in glucose metabolism and insulin sensitivity [178].

Nigella sativa oil (100 mg/kg, 10 weeks) decreased IL-6, reduced body weight by 18%, and improved mood scores in obese rats with depression [179]. Nguyen et al. [180] observed that curcumin (200 mg/kg) lowered cortisol, improved mood-related behavior, and modulated serotonin and dopamine in obese, stressed rats.

These results support translational research toward plant-based, multi-target interventions. For example, EGCG (300 mg/day) combined with a low-glycemic diet could be tested in obese individuals with mild depressive symptoms over 12–16 weeks, assessing outcomes such as body fat percentage, HDRS scores, insulin sensitivity, and BDNF levels.

In summary, natural compounds, such as curcumin, EGCG, anthocyanins, omega-3 fatty acids, and Nigella sativa oil, show synergistic anti-inflammatory, neuroprotective, and mood-regulating effects in vivo, supporting their use in integrated obesity and mental health strategies.

## 8. Personalized Diets Enriched with Bioactive Compounds and Probiotics in the Management of Obesity and Depression

Based on emerging evidence, individualized diets enriched with selected bioactive compounds offer promising adjunctive support for managing obesity and depression. Their synergistic actions modulate inflammation, neurochemical pathways, and metabolic functions. In their review, Lee et al. [181] emphasized the growing relevance of dietary bioactive compounds—including curcumin, epigallocatechin gallate (EGCG), and omega-3 fatty acids—in managing both obesity and depressive symptoms. These compounds exhibit anti-inflammatory, neuroprotective, and antioxidant properties while modulating cognitive functions and mood [181].

Clinical trials evaluating the efficacy of these compounds typically employed intervention periods ranging from 8 to 12 weeks. These involving curcumin (500–2000 mg/day), EGCG (200–400 mg/day), and omega-3 fatty acids (1–3 g/day) have reported modest weight reduction (1.5–3 kg), lower inflammatory markers (CRP, IL-6), and improved mood assessed by HDRS [181]. Mood improvement was commonly assessed using standardized tools such as the Hamilton Depression Rating Scale (HDRS). The synergistic action of these compounds is thought to result from their combined modulation of inflammation, neurogenesis, and neurotransmitter signaling. Nevertheless, larger and longer-duration clinical trials are warranted to determine optimal dosing strategies and confirm sustained efficacy [181]. Future directions should include the formulation of tailored nutraceuticals combining selected compounds (e.g., EGCG, curcumin, and anthocyanins) with proven safety profiles and bioavailability. These products may serve as adjunct therapies in lifestyle interventions or medical nutrition therapy (MNT) protocols for patients with obesity and comorbid depression.

The development of precision functional food products—such as anti-inflammatory meal kits or ready-to-drink probiotic beverages—could incorporate these compounds. Formulations targeting specific phenotypes (e.g., high BMI with elevated CRP and depressive symptoms) should be explored in personalized nutrition trials. Real-world applications might include lifestyle intervention programs that incorporate phytochemical-rich diets with app-based monitoring.

Preclinical studies confirm the efficacy of curcumin (200 mg/kg), resveratrol (50–100 mg/kg), and herbal extracts, such as ginseng and ashwagandha, in reducing fat mass, enhancing insulin sensitivity, and improving mood-related behaviors in rodent models [182,183].

The role of the gut microbiota in the pathogenesis of both obesity and depression is increasingly recognized. EGCG has been shown to favorably modulate gut microbiota composition, enhance SCFA production, and improve behavioral and metabolic parameters in HFD-fed rodents [184]. The intervention also enhanced short-chain fatty acid (SCFA) production and improved gut barrier integrity, thereby reducing systemic inflammation, a critical mechanism implicated in both disorders [184].

Recent studies highlight the synergistic benefits of combining bioactive compounds with probiotics. The co-administration of curcumin with *Lactobacillus rhamnosus* and *Bifidobacterium longum* enhanced weight loss and mood improvement in obese mice [185]. Similarly, a clinical trial combining ginger extract with probiotics led to reduced cortisol levels and improved psychological well-being in obese patients [186].

## 9. Gut Microbiota Modulation by Bioactive Compounds and Probiotics

In vitro fermentation models have shown that flavonoids from berries (e.g., anthocyanins from blueberries) and phenolic compounds from green tea can modulate gut microbiota by promoting beneficial bacteria, such as *Bifidobacterium* and *Lactobacillus* spp., while inhibiting pathogenic species [187,188]. These extracts also increased the production of short-chain fatty acids (SCFAs), particularly butyrate and acetate, which support gut barrier integrity and contribute to gut–brain axis signaling [188].

In vivo studies using rat models of diet-induced obesity revealed that turmeric and green tea extracts increased the abundance of SCFA-producing bacteria such as *Faecalibacterium prausnitzii*, while reducing levels of obesity-associated *Proteobacteria*. These microbiota shifts correlated with improved metabolic outcomes, including reduced fasting glucose, better insulin sensitivity, and improved lipid profiles [189].

SCFAs are key mediators of the gut–brain axis that affect neurobehavioral function and mood regulation [190]. Supplementation with plant extracts that enhance SCFA production has been shown to lower cortisol levels and reduce depressive-like behaviors in obese animal models [191]. For example, turmeric extract increased intestinal butyrate and acetate levels in obese rats, which coincided with improvements in behavioral tests related to mood [192].

Evidence also supports the synergistic effects of plant extracts and probiotics. In vivo studies in mice demonstrated that combining *Lactobacillus rhamnosus* with ginger extract resulted in greater reductions in body weight and improved metabolic markers compared to either treatment alone [193]. This combination enhanced the growth of SCFA-producing bacteria and improved gut barrier function by reducing intestinal permeability and inflammation, which are factors commonly associated with both obesity and depression [194].

## 10. The Gut–Brain Axis and Translational Potential Perspectives

The interaction among bioactive plant compounds, probiotics, and microbial metabolites plays a crucial role in the gut–brain axis, which is a key regulator of metabolic and neurobehavioral functions. Studies have shown that the modulation of gut microbiota and microbial products, such as SCFAs and tryptophan-derived indoles, can influence neurotransmission, neuroinflammation, and HPA axis function [195].

In a study by Li et al. [196], a combined intervention using probiotics (*Lactobacillus rhamnosus* and *Bifidobacterium longum*, 10^9^ colony-forming units (CFU)/day), prebiotics (fructooligosaccharides (FOS) and galactooligosaccharides (GOS)), and plant extracts (turmeric 200 mg/day and green tea 150 mg/day) improved behavioral and metabolic outcomes in animal models with obesity and depression. The treatment led to a ~25% reduction in cortisol levels, lower LDL and triglycerides, and reduced symptoms of anxiety and depression (assessed by open field and avoidance tests). These effects were attributed to the enhanced regulation of the HPA axis and neuroinflammation.

Similarly, Wang et al. [186] conducted a 12-week randomized controlled trial in 120 overweight and obese participants. The intervention included ginger extract (1 g/day) and probiotics (Lactobacillus acidophilus and Bifidobacterium bifidum, 10^9^ CFU/day). Participants who received the combined treatment showed significant improvements: ~25% cortisol reduction, ~3 kg weight loss, decreased IL-6 and TNF-α levels, and better scores on the Beck Depression Inventory-II and Perceived Stress Scale. Waist circumference and body mass index (BMI) also improved.

**Figure 6:**
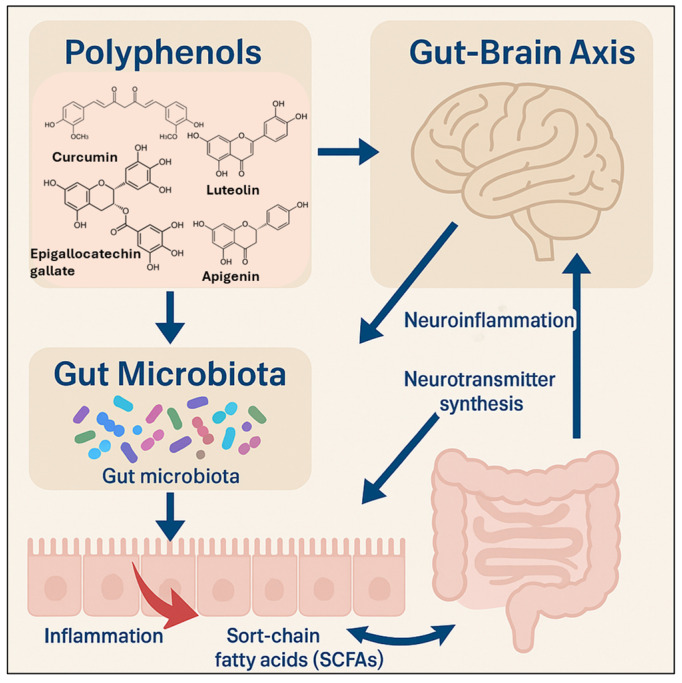
Synergistic interactions among plant bioactives, probiotics, and gut microbiota in modulating inflammation, metabolism, and neurobehavioral outcomes through gut–brain axis mechanisms.

These findings support the potential of combining specific plant-based compounds with probiotics and prebiotics to target both the metabolic and psychological pathways involved in obesity and depression. The gut–brain axis emerges as a promising therapeutic target for multi-component interventions (Figure 6) [186].

### Synbiotic-Based Strategies and Clinical Implementation

The gut–brain axis is increasingly recognized as a bidirectional communication system involving neural, immune, endocrine, and metabolic pathways. This axis plays a significant role in both the pathophysiology of obesity and depression. Alterations in gut microbiota composition—dysbiosis—have been linked to increased gut permeability, systemic inflammation, altered neurotransmitter production, and impaired HPA axis function [179,180].

Preclinical and human studies have shown that specific plant-derived polyphenols and flavonoids can modulate the composition and activity of the gut microbiota, favoring the growth of beneficial bacteria (e.g., *Bifidobacterium*, *Lactobacillus*) and suppressing pro-inflammatory taxa [181,182,183]. This modulatory effect is associated with the increased production of short-chain fatty acids (SCFAs), enhanced intestinal barrier integrity, and decreased circulating endotoxins (e.g., LPS).

Importantly, a promising translational strategy involves the combination of targeted probiotics with selected plant polyphenols in the form of synbiotic formulations. Such interventions could exert synergistic effects by modulating both local gut inflammation and systemic immune–neuroendocrine pathways. For instance, anthocyanin-rich extracts co-administered with *Lactobacillus rhamnosus* strains have been shown to reduce depressive-like behaviors and normalize corticosterone levels in animal models of stress-induced obesity.

From a clinical perspective, these insights could inform the development of next-generation personalized nutrition therapies, integrating gut microbiota profiling with tailored supplementation (e.g., polyphenols + probiotics) in individuals with comorbid depression and metabolic dysfunction. Pilot studies could include outcomes such as microbiota composition (16S rRNA), SCFA levels, CRP, IL-6, and validated mood scales (e.g., PHQ-9, BDI-II).

This microbiota-centered therapeutic approach aligns with the principles of precision medicine and supports the design of non-pharmacological, diet-based adjuncts to traditional care for mood and metabolic disorders.

## 11. Functional Diets as a Therapeutic Strategy

Recent clinical and preclinical evidence supports the potential of personalized diets enriched with plant-derived bioactive compounds and probiotics in managing obesity accompanied by depressive symptoms. Combinations of curcumin, EGCG, and omega-3 fatty acids have been shown to improve metabolic profiles, mood, and neuroendocrine regulation [181,182]. Similarly, supplementation strategies targeting the gut microbiota contribute to anti-inflammatory effects and the modulation of the hypothalamic–pituitary–adrenal (HPA) axis, with measurable benefits in both metabolic and psychological domains [186,196].

These findings highlight the potential of functional diets or nutraceuticals based on the synergistic actions of bioactive compounds and probiotics. Such interventions may downregulate inflammation, support neurotransmitter balance, regulate HPA activity, and improve energy metabolism—mechanisms relevant to both obesity and depression. While translational clinical trials remain limited, the emerging data point to a promising adjunctive strategy with fewer adverse effects compared to pharmacological monotherapies.

In summary, integrating phytochemicals, probiotics, and microbiota-targeted approaches may offer an effective and well-tolerated therapeutic pathway for patients with obesity and comorbid depression. Continued research into standardized formulations, optimal dosing, and long-term outcomes will be essential for advancing this functional nutrition strategy into clinical practice.

To accelerate translation into practice, public–private collaborations between academic centers, nutraceutical companies, and health systems could initiate pilot programs evaluating the impact of polyphenol- and probiotic-enriched diets. Digital health tools could monitor compliance, mood, and weight changes in real time, integrating these strategies into holistic obesity–depression management frameworks.

### Toward Clinical Applications and Functional Product Development

Although multiple in vitro and in vivo studies support the efficacy of bioactive plant compounds in mitigating obesity and depression, translation into clinical practice remains limited. To bridge this gap, structured clinical trials using standardized formulations, validated endpoints (e.g., anthropometric changes, inflammatory markers, and validated psychiatric scales), and well-defined patient groups (e.g., adults with BMI > 30 and elevated PHQ-9 scores) are urgently needed [160,161,162,163,164,165,166,167,168,169,170].

Based on the available evidence, several compounds reviewed in this manuscript—such as epigallocatechin gallate (EGCG), curcumin, resveratrol, genistein, and anthocyanins—are strong candidates for clinical testing as part of functional food interventions or nutraceutical formulations. Bioavailable doses (e.g., EGCG 200–400 mg/day; curcumin 500–1500 mg/day; and anthocyanin-rich extracts 300–500 mg/day) may be incorporated into comprehensive dietary strategies targeting comorbid metabolic and mood disorders.

Potential clinical trial pathways include:

- Phase I/II studies assessing the safety, tolerability, and preliminary efficacy of combined nutraceuticals (e.g., EGCG + curcumin + omega-3 fatty acids) in obese individuals with depressive symptoms;
- Randomized controlled trials (RCTs) evaluating synbiotic approaches (e.g., anthocyanins or flavonoids + *Lactobacillus rhamnosus* or *Bifidobacterium breve*) targeting inflammation and the gut–brain axis;
- Comparative studies testing phytochemical-enriched diets versus standard pharmacotherapy in patients with mild-to-moderate depression and insulin resistance.

From a functional food development perspective, the creation of microbiota-modulating, polyphenol-enriched products (e.g., flavonoid-fortified snack bars, anthocyanin-based drinks with probiotics) represents a promising translational route. These innovations could be informed by nutrigenomics, metabolomic screening, and gut microbiota profiling, supporting personalized, anti-inflammatory, and mood-regulating dietary protocols.

Furthermore, growing patient interest in dietary interventions reinforces the need to develop evidence-based nutritional guidelines for clinical use. Future research should also prioritize long-term safety, efficacy in diverse populations, bioavailability enhancement strategies (e.g., nanoencapsulation and emulsifiers), and regulatory compliance with EFSA and FDA standards [180,181,182,183,184].

## 12. Pharmacokinetics and the Potential Side Effects of Selected Phyto-Chemicals: Potential Adverse Effects of Major Compounds (Curcumin, EGCG, and Resveratrol)

In the context of the expanded use of phytochemicals, such as curcumin, EGCG and resveratrol, it is important to understand their pharmacokinetics and the potential risks associated with their use. The following is a detailed review of available data based on the scientific literature.

### 12.1. Adverse Effects of Resveratrol

Resveratrol (RE) is a widely studied polyphenol with antioxidant, anti-inflammatory, cardioprotective, and neuroprotective properties. Despite promising preclinical outcomes, clinical evidence on its effectiveness remains mixed, and studies on its long-term safety are limited [197].

One of the main challenges in using RE therapeutically is its poor oral bioavailability, which is largely due to its low water solubility (<0.05 mg/mL), rapid metabolism, and extensive conjugation. Although about 70% of ingested RE is absorbed, plasma levels of the unmetabolized compound remain extremely low (<5.0 ng/mL after a 25 mg dose) [197]. Zupančič et al. [198] reported pH-dependent solubility, with optimal values around a pH level of 1.2 and reduced solubility above a pH level of 7.4. RE also shows limited stability at neutral or alkaline pH and degrades under light and oxygen exposure [198,199,200,201].

In vivo, RE binds to albumin, LDL, and integrins, potentially enhancing its tissue distribution and bioactivity [202]. Its metabolites—glucuronides and sulfates—circulate longer than the parent compound and may act as reservoirs for sustained release [203,204].

Toxicological concerns primarily arise at high doses. RE may interfere with cytochrome P450 enzymes, leading to drug interactions and endocrine disruption. High concentrations have been associated with thyroid nodules, pro-oxidant activity, and apoptosis in normal cells via reactive metabolites like o-quinones [205,206,207].

Clinical reports include side effects such as gastrointestinal distress (nausea, diarrhea, and rectal pruritus), elevated liver enzymes (e.g., ALT), leukopenia, and hypersensitivity reactions at doses between 2 and 5 g/day [208,209,210]. Patients with thyroid disorders may be at increased risk of adverse outcomes.

Animal studies confirm dose-dependent toxicity. Doses ≥50 mg/kg in rodents caused nephropathy, inflammation, and mortality, while doses ≤25 mg/kg appeared safe [211,212]. In zebrafish embryos, high doses of RE led to developmental abnormalities and cardiovascular defects [213,214].

In conclusion, while resveratrol is considered safe at moderate levels, high doses or long-term use may lead to adverse effects, including oxidative stress, organ damage, and hormonal dysregulation. Further clinical and toxicological studies are essential to define safe therapeutic ranges and understand their systemic impact [203,204,215,216,217,218].

### 12.2. Adverse Effects of Epigallocatechin Gallate 

Adverse effects of epigallocatechin gallate (EGCG) include a range of toxic effects that can occur with excessive intake or as a result of long-term use of the compound. The literature emphasizes that high doses of EGCG can cause serious liver damage, which has been confirmed in both animal studies and clinical reports. Clinical signs associated with hepatotoxicity include elevated levels of liver enzymes such as transaminases (ALT and AST), as well as the presence of jaundice, abdominal pain, and increased risk of jaundice [217,218,219]. Evidence suggests that the liver is a major target organ for the toxic effects of EGCG, mainly due to its role in metabolizing the compound, as evidenced by the high concentrations of EGCG detected in liver tissue after oral administration [220]. At the cellular level, EGCG exhibits cytotoxic effects on hepatocytes, causing damage to cell membranes and mitochondrial potentials, resulting in reduced cell function and cell death [221,222]

Experimental studies have shown that high doses of EGCG can induce oxidative stress, leading to lipid peroxidation, as evidenced by increases in markers such as MDA and 4-HNE in liver samples [218]. These mechanisms involve activation of the Nrf2-ARE pathway, which under conditions of EGCG excess may act as a defensive response, but at the same time indicates overloading of the body’s an-oxidative systems [223].In addition, excess EGCG can lead to the two-boosting of thiol conjugates, such as EGCG-2′-cysteinyl, which are biomarkers of oxidative stress and chemical damage caused by pro-oxidant compounds [224,225].

The pharmacokinetics of EGCG are characterized by low bioavailability, ranging from 0.2% to 2%, meaning that most of the ingested compound is degraded in the gut by the microbiota or metabolized in the liver [226,227].This indicates that even at high doses, which may exceed safe levels, a small amount of active EGCG can accumulate in the body, increasing the risk of side effects. The safety of EGCG use is therefore related to the dose, the time of administration and the individual’s ability to metabolize the compound, and exceeding established tolerance levels can result in serious health effects, mainly related to liver and kidney function [228,229].

In addition to its effects on the liver and kidneys, EGCG also shows adverse effects on other organs and tissues, mainly due to its pro-oxidant properties. For example, animal studies conducted by Chu et.al. [230] showed that EGCG can harm eye tissues because it causes oxidative stress in various parts of the eye, such as the plasma, aqueous fluid, vitreous body, cornea and retina. High doses of EGCG increase the level of the enzyme SOD1, while suppressing the expression of catalase (CAT), which leads to an excess of reactive oxygen species and oxidative stress in the retina. As a result, the neutralization of ROS to H_2_O_2_ is faster, but the decrease in CAT activity hinders the removal of excess H_2_O_2_, exacerbating oxidative stress [230].

Oxidative stress can damage pancreatic islet β cells and reduce tissue insulin sensitivity, which may contribute to the development of type 1 diabetes [231]. In rats with streptozotocin (STZ)-induced diabetes, four-day administration of EGCG at 5 mg/kg/intramuscularly aggravated β-cell damage and reduced their response to high glucose concentrations. In this context, vitamin E, which has antioxidant activity, blocked these deleterious effects, suggesting that EGCG acted as a pro-oxidant [232].

In addition, in vitro studies have shown that EGCG is toxic to embryos. At concentrations ranging from 25 to 50 μM, EGCG markedly increased apoptosis in mouse blastocysts, reduced cell number, and interfered with subsequent embryonic development [83]. This damage was related to intrinsic apoptotic signaling processes, as specific inhibitors of caspase-9 and caspase-3 effectively blocked these effects, indicating a pro-oxidant mechanism of EGCG leading to apoptosis.

In terms of effects on DNA, EGCG has shown the ability to damage genetic material. In healthy human lymphocytes, concentrations of 1 to 100 μM caused DNA strand breaks [233], and other studies have confirmed that in a similar dose range (10–100 μM), EGCG causes DNA damage, which is related to the substance’s prooxidant effects [84]. It is worth mentioning that despite these actions, EGCG does not show clastogenic effects in in vivo studies [234].

In addition, in fat cells, EGCG induces oxidative stress, which can have negative health effects by interfering with hormone function in adipocytes [235]. In conclusion, although EGCG is mainly known as an antioxidant, its pro-oxidant effects can lead to cellular, DNA, and tissue damage, which is important in the context of the potential adverse effects of this substance. Excessive consumption of epigallocatechin gallate is associated with the risk of hepatotoxicity, nephrotoxicity, and other adverse effects, which calls for caution and monitoring of doses in the context of use as a dietary supplement.

### 12.3. Adverse Effects of Curcumin

Although curcumin is widely considered safe and is widely used as a dietary ingredient and supplement, there is evidence of its potential toxicity at certain conditions and doses. In vitro and in vivo studies have shown that curcumin can induce DNA damage and chromosomal aberrations at concentrations similar to those that show beneficial effects (2.5–5 µg/mL). Such effects, including induction of mutations and genetic damage, have been linked to carcinogenesis, raising concerns about the safety of long-term use [230,236,237,238,239,240,241]. 

Long-term studies in animal models have shown that intake of curcumin in high doses can lead to pathological changes such as inflammation, hyperplasia and ulceration in the gastrointestinal tract, as well as hyperplasia of thyroid cells and increased risk of cancer in some organs [242]. Notably, consumption of diets containing turmeric extract (containing 79–85% curcumin) over a period of up to 2 years increased the incidence of tumors, suggesting a potential mutagenic or tumor-promoting effect [242,243].

Curcumin can generate reactive oxygen species (ROS) in high concentrations, which contributes to oxidative stress and DNA damage. In addition, the presence of a 2,4-en-xetone group in its chemical structure enables Michael addition-type reactions that can modify key proteins and enzymes, including topoisomerase II, p53 and antioxidant enzymes such as thioredoxin-reductase, which can lead to uncontrolled cancer cell growth [244,245,246,247,248].

Curcumin shows poor bioavailability after oral administration due to its intensive metabolism in the intestines and liver. Studies have shown that after large doses (e.g., 10–12 g) in studies on healthy volunteers, only one person could detect the presence of free curcumin in plasma at nanomolar levels, which is well below the concentrations needed to induce cytotoxic effects [249,250]. In addition, curcumin is metabolized mainly to glucuronides and sulfates, which limits its availability in some tissues and organs [249,251,252]. 

Moreover, curcumin has the ability to inhibit drug-metabolizing enzymes such as cytochrome P450, glutathione-S-transferase and UDP-glucuronosyltransferase. This can lead to increased concentrations of certain drugs in the body, which increases the risk of side effects [253,254,255,256,257,258].

According to reports from international organizations such as JECFA and EFSA, the safe daily dose of curcumin is up to 3 mg per kilogram of body weight. In studies on healthy individuals using doses of 0.45 to 3.6 grams per day for up to four months, mainly mild side effects such as nausea, diarrhea, headaches and increased liver enzyme levels were reported [259,260]. At higher doses, especially above 8 g per day, more serious effects may occur, although the data are limited and long-term safety is not yet fully confirmed [260].

In conclusion, despite the widespread belief in curcumin’s safety as a dietary supplement, there is growing evidence of its potential harm, especially at high doses and long-term use. Cases of DNA-damaging effects, tumor promotion and serious pathological changes in animal models indicate that its use must be cautious and preceded by thorough safety studies. In addition, its low bioavailability limits therapeutic efficacy, necessitating strategies that increase absorption, but which may also increase the risk of side effects.

Summarizing the most significant adverse effects on the body, it should be pointed out that curcumin can induce DNA damaging and chromosomal aberrations at the in vitro and in vivo levels, which may have oncogenic implications [236,237,238,239,240,241]. 

Long-term animal studies suggest that high doses can lead to pathological changes and increase the risk of cancer [242,243]. Mechanisms of deleterious effects include ROS generation and Michael addition-type reactions that can modify key proteins and enzymes [244,246,247,248].

In addition, low bioavailability after oral administration limits the achievement of effective tissue concentrations [249]. Curcumin can inhibit drug-metabolizing enzymes, which increases the risk of pharmacological interactions [253,254,255,256].

In addition, long-term use of high doses (e.g., more than 8 g per day) can lead to side effects such as nausea, diarrhea, increased liver enzymes, and iron deficiency [260,261].

## 13. Limitations

This scoping review has several limitations. First, the review was not registered in a systematic review registry, which may limit transparency compared to pre-registered protocols. Second, although the PRISMA guidelines were followed, no formal assessment of risk of bias was conducted for the included studies, as the focus was on mechanistic diversity and exploratory synthesis rather than quality-weighted meta-analysis. Third, only studies published in English were included, which may introduce language bias. Additionally, the inclusion of heterogeneous study types (in vitro, in vivo, and clinical) without stratification may affect the comparability of findings. Lastly, publication bias could not be excluded, as studies with negative results may have been underrepresented.

## 14. Conclusions

The comorbidity of obesity and depression represents a major clinical and public health challenge, which is characterized by overlapping mechanisms such as chronic inflammation, oxidative stress, neurotransmitter imbalances, and gut–brain axis disruption. This review underscores the therapeutic potential of plant-derived bioactive compounds—particularly polyphenols, flavonoids, alkaloids, and betalains—in targeting both metabolic dysfunction and mood disorders.

The key mechanisms of action include the reduction of neuroinflammation, the modulation of serotonergic and dopaminergic pathways, the enhancement of neurotrophic factors (e.g., BDNF), the suppression of adipogenesis, and the improvement of insulin sensitivity. Compounds such as quercetin, genistein, EGCG, curcumin, and anthocyanins have shown promising results in preclinical models and emerging evidence from human studies.

Incorporating these phytochemicals into functional diets or adjunct therapies may offer a safe, integrative strategy for patients with coexisting obesity and depressive symptoms. However, to facilitate clinical translation, future research should prioritize standardized dosing, long-term safety assessments, and synergistic combinations, especially those involving microbiota-targeted interventions.

Ultimately, integrating phytotherapy with personalized nutrition and microbiota modulation may redefine future therapeutic approaches for obesity-related mood disorders.

## Data Availability

Not applicable.
